# Yolk proteins of the schistosomiasis vector snail *Biomphalaria glabrata* revealed by multi-omics analysis

**DOI:** 10.1038/s41598-024-52392-x

**Published:** 2024-01-20

**Authors:** Mohamed R. Habib, Lijing Bu, Marijan Posavi, Daibin Zhong, Guiyun Yan, Si-Ming Zhang

**Affiliations:** 1 Department of Biology, Center for Evolutionary and Theoretical Immunology, University of New Mexico, Albuquerque, NM 87131 USA; 2 Program in Public Health, College of Health Science, University of California, Irvine, CA 92697 USA

**Keywords:** Developmental biology, Evolution, Molecular biology, Zoology

## Abstract

Vitellogenesis is the most important process in animal reproduction, in which yolk proteins play a vital role. Among multiple yolk protein precursors, vitellogenin (Vtg) is a well-known major yolk protein (MYP) in most oviparous animals. However, the nature of MYP in the freshwater gastropod snail *Biomphalaria glabrata* remains elusive. In the current study, we applied bioinformatics, tissue-specific transcriptomics, ovotestis-targeted proteomics, and phylogenetics to investigate the large lipid transfer protein (LLTP) superfamily and ferritin-like family in *B. glabrata*. Four members of LLTP superfamily (BgVtg1, BgVtg2, BgApo1, and BgApo2), one yolk ferritin (Bg yolk ferritin), and four soma ferritins (Bg ferritin 1, 2, 3, and 4) were identified in *B. glabrata* genome. The proteomic analysis demonstrated that, among the putative yolk proteins, BgVtg1 was the yolk protein appearing in the highest amount in the ovotestis, followed by Bg yolk ferritin. RNAseq profile showed that the leading synthesis sites of BgVtg1 and Bg yolk ferritin are in the ovotestis (presumably follicle cells) and digestive gland, respectively. Phylogenetic analysis indicated that BgVtg1 is well clustered with Vtgs of other vertebrates and invertebrates. We conclude that, vitellogenin (BgVtg1), not yolk ferritin (Bg yolk ferritin), is the major yolk protein precursor in the schistosomiasis vector snail *B. glabrata*.

## Introduction

In oviparous animals, a large amount of yolk proteins is selectively accumulated in oocytes for oocyte growth and embryonic development^1–6^. Yolk platelets or granules normally comprise several different yolk proteins in variable amounts. The most abundant one is the major yolk protein (MYP). In most oviparous animals, vitellin is a major yolk protein that provides nutrients, including lipids to the developing oocytes. Vitellin is proteolytically cleaved from its precursor, vitellogenin (Vtg). Therefore, conventionally, vitellin and Vtg are called MYP and MYP precursors, respectively^1–4^.

Vtgs are members of the large lipid transfer (LLT) protein (LLTP) superfamily, which includes vertebrate apolipoprotein B (ApoB), insect apolipophorin II/I precursor (ApoLp-II/I), Vtg, and microsomal triglyceride transfer protein (MTP)^7–9^. Each family member binds lipids, although their functions differ^10^. For example, Vtg binds only a small quantity of lipids, predominantly phospholipids. MTP is found in both vertebrates and invertebrates and aids in the synthesis of ApoB and Vtg^11,12^. Structurally, all members of the LLTP superfamily share a conserved lipoprotein N-terminal domain (LPD_N) but differ in the downstream regions of the proteins. Apos, including vertebrate ApoB and insect ApoLp-II/I possess LPD_N and DUF1081, but either or both DUF1943 and Von Willebrand factor D (VWD) may not be present. Vtgs consist of LPD_N, DUF1943, and VWD, whereas MTPs have only LPD_N domain^8,13^. It was believed that LLTPs had emerged from an ancestral Vtg gene before the divergence of cnidarians and bilaterians^7,8^.

Yolk proteins, particularly Vtgs, have been extensively studied in oviparous vertebrates and insects due to their essential role in reproduction^6,8,9,14^. Information concerning yolk proteins in Mollusca, the second-largest phylum of invertebrate animals after the Arthropoda, is still scarce and often speculative. An early work published in 1979 has surprisingly revealed that the secreted form of ferritin, an iron storage protein, serves as MYP, in the two species of gastropod snails, *Planorbarius corneus* and *Lymnaea stagnalis*^15^. There was no evidence suggesting the presence of vitellin or Vtg in this group of gastropod snails, although Vtg was well-known as MYP at that time^16–18^. Since the discovery of the unique ferritin protein as MYP in animals, referred to as yolk ferritin or vitellogenic ferritin, was strikingly different from the classic notion, with respect to the nature of MYP (i.e., Vtgs) and the general biological function of the well-characterized vertebrate ferritins (i.e., cytoplasmic ferritins), subsequent intensive investigations using multiple approaches, including electron microscopy, crystallography, immuno-chemical assays, and biochemical analysis, have been performed^18–24^. These studies have collectively demonstrated that yolk ferritin is synthesized in the digestive gland, secreted into the hemolymph, and transported into the oocytes by oocyte receptor-mediated endocytosis^24,25^.

The freshwater gastropod snail *Biomphalaria* *glabrata* is not only an essential intermediate host of human schistosomiasis, a snail-borne parasitic disease that currently afflicts more than 251 million people worldwide, but also a model snail species used for schistosomiasis research^26–29^. Gastropod snails are hermaphroditic and possess similar anatomical structures of reproductive organs (ovotestis) and gametes^16,30^. These characteristics make transferring or adapting basic biological knowledge from one species to another possible and valuable as they are invertebrate models for neurobiology and developmental biology and obligate vectors for parasitic diseases such as schistosomiasis and fascioliasis^28,31–33^. Regarding yolk proteins, previous findings from research on *B. glabrata* and *P. corneus* led to the hypothesis that yolk ferritin-like protein, but not Vtg, is MYP in *B. glabrata*^17,24^, although investigation of yolk proteins has not been conducted on any *Biomphalaria* snails. This assumption may be due to the fact that *B. glabarata* is closely related to the two gastropod species described above, as both *B. glabrata* and *P. corneus* belong to the same Planorbidae family. Although, *L. stagnalis* belongs to another family (Lymnaeidae), the three snail species belong to the order Lymnaeoidea (Panpulmonata, Hygrophila)^34,35^.

To test this hypothesis and enable a better understanding of *Biomphalaria* reproductive biology, we conducted a comprehensive analysis of genomics, transcriptomics, proteomics, and phylogenetics focusing on two types of proteins (i.e., LLTPs and ferritins) because member(s) of either one could serve as MYP in *B. glabrata. *Remarkably, our data suggest that the MYP precursor of *B. glabrata* is Vtg, the common MYP of oviparous animals^1–3^, but not yolk ferritin, as discovered in the gastropod snails *P. corneus* and *L. stagnalis*^15^. This finding improves our understanding of the reproductive biology of planorbid snails. In addition, given the importance of *Biomphalaria* spp. as intermediate hosts for *Schistosoma mansoni*, it could be useful for the development of more specific snail-based biocontrol programs of schistosomiasis. Disruption of key gene(s) involved in egg development via gene drive may lead to the control of snail populations in the field, thus reducing schistosomiasis transmission in endemic areas ^36–38^.

## Methods

### Snails

Colonies of *B. glabrata* M line have been maintained at the Center for Evolutionary and Theoretical Immunology (CETI) of the University of New Mexico (UNM), United States^39^. The culture of snails has been performed routinely, according to Lewis et al.^40^.

### Identification of LLTP and ferritin-like genes and analysis of their expression

Although we are interested in Vtg-like yolk proteins, we cannot rule out the potential role of other members of LLTP in vitellogenesis because the definition and function of LLTP members still need to be clarified in invertebrates. For example, the major egg yolk precursor protein Vtg in decapod crustaceans was later found to be homologous to insect ApoLp-II/I and vertebrate ApoB^13^. Therefore, it is necessary to investigate all members of the LLTP in *B. glabrata*.

Vtg of the abalone *Haliotis discus hannai* (GenBank accession number BAF98238.1)^41^ and yolk ferritin of the snail *L. stagnalis* (GenBank accession number X56779.1)^42^ were used as query to BlastP iM line genome sequence, followed by manual curation^39^. Domain prediction was made using SMART program (http://smart.embl-heidelberg.de)^43^, and signal peptide (SP) was predicted using PrediSi—Prediction of Signal Peptides and their Cleavage Positions (http://www.predisi.de)^44^.

Heatmap analysis was used to examine the differential expression of four LLTP members and five ferritin-like genes identified in *B*. *glabrata*. RNA sequencing (RNAseq) data from 11 tissues of *B. glabrata* BBO2^45^ were downloaded from the NCBI Sequence Read Archive (SRA) with SRA run accession numbers SRR1509459—SRR1509470 and SRR1509473. The raw reads of each tissue were trimmed based on the base calling quality and read length using Trimmomatic v0.36^46^. Clean reads were mapped to the reference *B. glabrata* BB02 genome using STAR v2.2.1^47^. Gene counts measured in reads per kilobase of transcript per million reads mapped (RPKM) values were calculated using StringTie v1.3.5^48^. The blocks in the heat map were colored based on Z scores, calculated from transcripts per million (TPM). The Z score was determined by subtracting the mean TPM across all organs from the TPM of each tissue, then dividing by the standard deviation of TPM across all tissues examined. This normalization method allowed for comparing TPM values of each gene across different tissues by transforming them into comparable Z scores. Heatmap figures were generated using the R package heatmap 1.0.12.

### Proteomic analysis of ovotestes

The healthy *B. glabrata* M line snails were used for the study. Dissection of ovotestes was conducted under stereomicroscopy. To prevent the contamination of non-ovotestes tissues, the ovotestes were carefully dissected and quickly washed with phosphate buffer saline (PBS) solution three times. After cleaning using Kimwipes, the ovotestes were then placed into a 1.5 ml tube. Ovotestes extracted from 5 mature M line snails (~ 12 mm in shell diameter) were pooled and sent (on dry ice) to Creative Proteomics (www.creative-proteomics.com) for label-free 5-fraction LC–MS/MS analysis. The ovotestes were lysed, precipitated, and dissolved in 2 M urea aqueous solution. The protein sample was reduced by 10 mM DL-dithiothreitol (DDT) at 56 °C for 1 h, followed by alkylation with 50 mM iodoacetamide (IAA) for 1 h at room temperature in the dark. After adding ammonium bicarbonate (ABC) to the sample (at a final concentration of 50 mM ABC at pH 7.8), the sample was digested by trypsin (37 °C, 15 h). The digested peptides were purified with a C18 SPE column (Thermo Scientific, Waltham, MA). The sample was fractionated into 5 fractions using HPLC, dried in vacuum, and stored at -20 °C. The ultimate 3000 nano UHPLC system (Thermo Scientific) was applied with a loaded volume of 1 mg sample. The full scan was performed between 300–1,650 m/z at a resolution of 60,000 at 200 m/z. The automatic gain control target for the full scan was set to 3e6. The MS/MS scan was operated in Top 20 mode using the following settings: resolution 15,000 at 200 m/z; automatic gain control target 1e5; maximum injection time 19 ms; normalized collision energy at 28%; isolation window of 1.4. Th; charge state exclusion: unassigned, 1, > 6; dynamic exclusion 30 s. Raw MS files were analyzed and searched against the Crotalus protein database based on the species of the samples using Maxquant (1.6.2.6). The parameters were set as follows: the protein modifications were carbamidomethylation (C) (fixed), oxidation (M) (variable); the enzyme specificity was set to trypsin; the precursor ion mass tolerance was set to 10 ppm, and MS/MS tolerance was 0.5 Da. Relative quantification was applied for estimating the abundance of identified proteins. The protein intensity was determined based on MS peak area, rather than absolute concentration. The percentage of each protein was calculated based on protein intensity value.

### Phylogenetic analysis of LLTP members and ferritin-like proteins

The BlastN search was applied to collect LLTP members and ferritin-like sequences from NCBI databases (https://www.ncbi.nlm.nih.gov), and their domain combinations were analyzed using SMART program^43^. After the collection of the sequences was done, the conserved LLT modular motifs of LLTPs, as described previously^7,10,13^, were extracted for alignments and subsequent phylogenetic analysis, whereas for ferritin-like proteins, the conserved ferritin domains were applied^49^. Molecular Evolutionary Genetics Analysis (MEGA11 version)^50^ was used for phylogenetic analysis. Amino acid (aa) sequences were aligned using ClustalW built in the MEGA11 program. The evolutionary tree was inferred by using the Maximum Likelihood method (ML) and JTT matrix-based model^51^ with a bootstrap value of 1,000.

## Results

### Genomic and transcriptomics analyses of members of the LLTP gene subfamily

**Figure 1:**
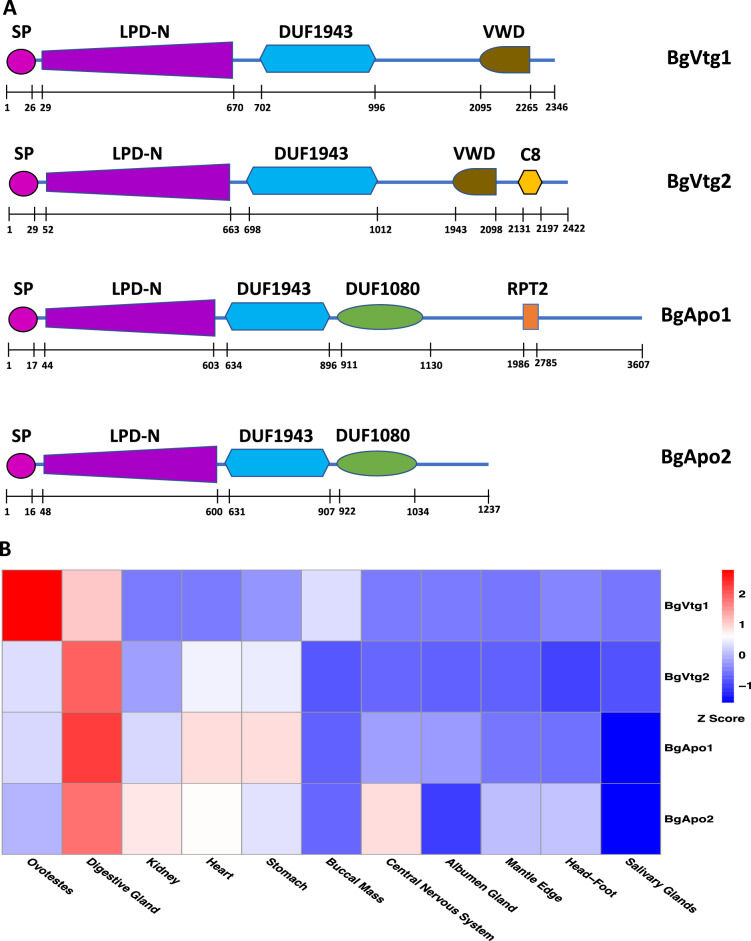
(**A**) A schematic diagram showing the modular architecture of 4 LLTP proteins, BgVtg1, BgVtg2, BgApo1, and BgApo2 (not drawn to scale). RPT: Internal Repeats. (**B**) A heatmap showing the relative expression of 4 LLTP proteins, BgVtg1, BgVtg2, BgApo1, and BgApo2 in 11 tissues of *B. glabrata*.

Four LLTP-encoding genes were identified. Based on the criterion proposed^8^, two of them belong to Vtgs, which were assigned BgVtg1 and BgVtg2 (Fig. 1, Supplementary Fig. 1). The overall aa identity between BgVtg1 and 2 was low (~ 45%), implying they are functionally different. A slight structural difference between the two BgVtgs is that BgVtg2 (2,422 aa) possesses a C8, a domain rich in cysteine residues at the C-terminus, but BgVtg1 (2,346 aa) lacks that domain (Fig. 1A,B). In addition, we also identified two Apo-like genes named BgApo1 and BgApo2. The modular architecture between the two is the same, but the size of Apo1 (3,607 aa) is much larger than that of BgApo2 (1,237 aa) (Fig. 1, Supplementary Fig. 1).

Heatmap analysis of RNAseq data collected from 11 tissues showed that the highest abundance of *BgVtg1* transcripts was observed in the ovotestis. In contrast, the highest abundance for the three other member transcripts (*BgVtg2, BgApo1, and BgApo2*) was found in the digestive gland (Fig. 1B).

### Genomic and transcriptomic analyses of members of the ferritin-like gene family

**Figure 2:**
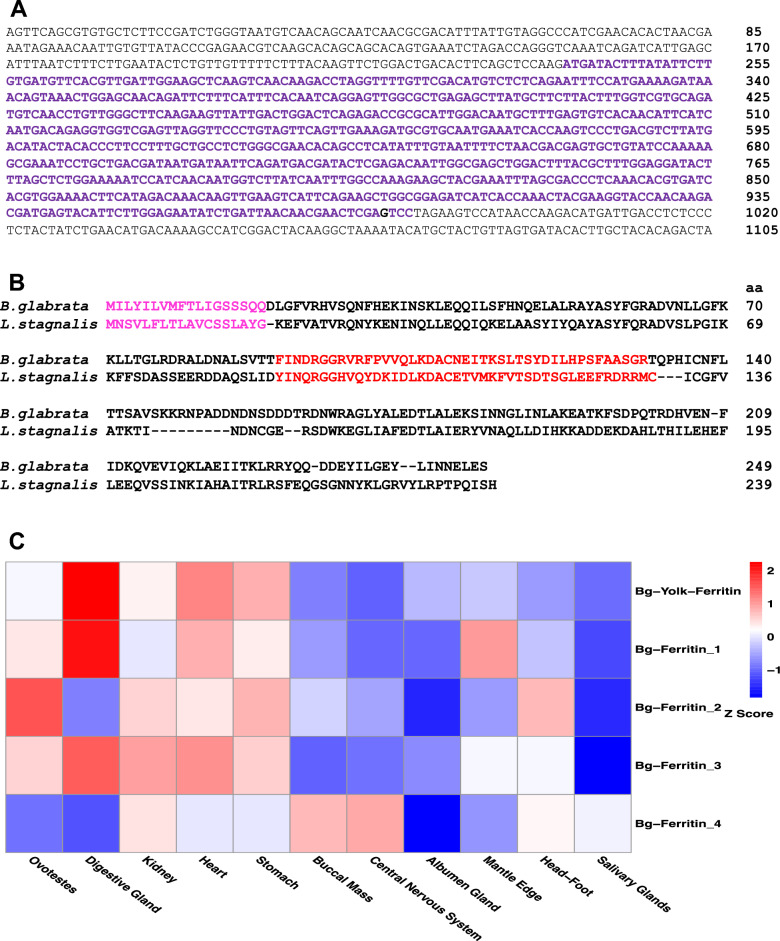
(**A**) A mRNA sequence of Bg yolk ferritin. Nucleotides in purple color are coding sequences. (**B**) A comparison of the deduced amino acid (aa) sequences of yolk ferritin between *B. glabrata* and *L. stagnalis*. Sequence in pink color is single peptide (SP) and sequence in red is the 42-aa insert sequence. (**C**) A heatmap showing relative expression of the yolk ferritin and four cytoplasmic ferritins in 11 tissues of *B. glabrata*.

We were interested in ferritins because yolk ferritins have been proposed as a MYP in gastropod snails. One *yolk ferritin* (secreted ferritin) gene and 4 soma ferritins (cytoplasmic ferritins) were identified in *B. glabrata* genome. Yolk ferritin and soma ferritins were designated Bg yolk ferritin and Bg ferritins, respectively. The Bg yolk ferritin protein consists of an 18-aa SP and a 231-aa mature peptide (Fig. 2A,B). To confirm the authenticity of the yolk ferritin, we compared Bg yolk ferritin to the homology of *L. stagnalis* and showed that Bg yolk ferritin shares multiple features with that of *L.* *stagnalis* (Fig. 2B). First, its 5’ untranslated region (5’UTR) does not contain an iron responsive element (IRE)^52^, a hallmark of classic ferritins, implying that regulation of the yolk ferritin is not controlled by iron (Fig. 2A). Second, both yolk ferritins from the two snail species have a 42-aa insertion^42^, in which no homolog has been documented in any other protein such as classic ferritins (Fig. 2B). Third, both yolk ferritins possess a SP, suggestive of a secreted protein (Fig. 2B; Supplementary Fig. 1).

Our heatmap revealed the highest abundance of Bg *yolk ferritin* transcripts was detected in digestive glands, with very minor or no expression in the other tissues, a similar pattern observed in *L. stagnalis*^42^ (Fig. 2C). For soma ferritins, their expression patterns were quite different. The highest expression of ferritin 2 gene was found in ovotestes. However, the pattern of ferritin 1 and 3 expression was like that of yolk ferritin (Fig. 2C).

### Proteomic analysis of ovotestes

A total of 4,729 proteins were identified in pooled ovotestis samples of *B. glabrata* using 5-fraction MS/MS analysis. We focused on the LLTP and ferritin-like proteins because they are likely the primary yolk proteins, the subject of this study. The results showed that BgVtg1 had the highest abundance among all LLTP and ferritin-like proteins, followed by Bg yolk ferritin. The complete order of relative abundance of LLTP or ferritin-like proteins and peptides related to each protein are listed in Table 1.

**Table 1:** Identification of peptides derived from LLTP members and ferritin-like proteins in the ovotestis of *B. glabrata*.

Protein	Relative abundance rank	Abundance percentage (%)	Number of peptides	Peptide sequence
BgVtg1	11	1.0154	85	ALSNSGLPHAVPIFNK, ALVEFAMPIQK, ANSESFNLK, ATPTETLINK, CAIAFNILTLK, DLQYSVSK, DNPLTTGAVVK, DTLSATWQK, DYTQCLER, EAISMHQLINK, EAISMHQLINKFEDVTER, EEYVYEYNTQILTGLPLHSSIHSGYR, ELTGSESTVIVESLTTPFGFDYENGQVK, ELTGSESTVIVESLTTPFGFDYENGQVKR, EMEWSANAK, ENQESIIK, EPDLEVYSVDAIISQLK, ESLVHLGVVVEMLSVSPR, EWQMVTEFR, EWQMVTEFRK, FEDVTER, FYDLNVLGYK, GFVSLFEIK, GGMPAAHLYLR, GIILSLDAST***Q***K, GSVSLSVEPSIFEDER, GVMEIYGSMGLDAHYMK, IEQLMLTLQTENSEALVQK, IESLDLGQR, IFEVNFAYNK, ILANHEPHLYFR, ILAVLGHR, ILFNVLPHVGTEAAVNQVLDICSQDLDFEK, IQSTMNFDLDEIIK, IQSTMNFDLDEIIKEK, ITSAFI***R***, IVFSPYLNK, IVNSFDK, KEMEWSANAK, KGGMPAAHLYLR, KSAIDIPDVFSLK, KSQVEALWSR, KVSASLDFRPK, LFGPQGLLTMNK, LLDNLVPEQLYMVMDK, LLDNLVPEQLYMVMDKVEVSK, LLVSLNTYTTK, ***L***MGHELR, LTHSLNQTEQHAR, LVSIVPELNKPLVK, LVSLHEK, MLLDVTSASLSEQELPIYNELK, MTLPTEAGYPVSLK, NIVDTINELVVSGSK, NIVLTQVQNIQNLMK, PVYLQTK, QAADNNLNIASR, QPITQIEAHENLIPEEYLK, QQSCDIELIK, QTAYLSLGSLGYK, QVFTL***S***LSDNEEK, RGFVSLFEIK, SAIDIPDVFSLK, SCKEPDLEVYSVDAIISQLK, SFTNLVTK, SKVNVQ***F***ENIR, SPMPDYTPK, SQVEALWSR, SSHQASNYLLHSTLLSEINELNYPAMMR, STSPQEMIEDIFK, SVDCQDPFELSK, TLLAVYNDKTEVNEVR, TMYYLMNTK, TTIPLHHVPETR, VGFVGQLPYQFDWQNPLHIMGGK, VLSVTSIGSMNR, VNVQFENIR, VRDYTQCLERPVYLQTK, VSASLDFR, VSASLDFRPK, VSLIMSLPSK, VTNSFNQEETSYSDPVDLDKEAISMHQLINK, YDGFFPIGHSK, YLDLSEYISSLMANGK, YLIEVYR
BgApo1	565	0.0313	80	AMAADAFR, DEYGVTVQTK, DISSFLVETLK, EHAFELDIK, ELNFEIDHTLDTDLSTTVR, ELSIEIEPPKDK, FDTSFNAQYGDYER, FQYAPSTTYIYDYVVDTETTMAGATEDSAK, GLLSAFQNSMANLDSNDR, GNTIFEGTTAFSYNSDSR, GPSDSLVVSLNHEITGDK, HIYVPFSNDQSGGK, IDFDGSLNAPSIELIELDTK, IIDLDVDYR, IILNVPLPMALER, IISDIQSLPIIR, IISQVPEQLK, IQTPSAVLLSLTGTGDYK, ISDAALTFTLVKPK, ISHSEAFAK, IVQLLEEEKDEQLGSYITSHLNNLK, IYWDNKEDSR, KFQYAPSTTYIYDYVVDTETTMAGATEDSAK, KPWPQEYIVIADLKEDK, LDLIYLMK, LDLTLVHPDR, LFGNELFFQHFSGVEGLLK, LGYNTVSTDFSNLMK, LLGPYFSGNK, LNLGLVADSDVISGLDSPLPR, LSDSINNANQFIQK, LSLDVNQDK, LTFSHETTR, LTLNTPLR, MESATQTLK, MFSELVTVLR, MISADVTIGYAPK, MNLNVETPYR, MNYALGTEEHK, MSWPTDSLETTWTHDNTDTR, NDAYDVISDVNLDGYDPIK, NENKFDTSFNAQYGDYER, NFQVNPEVK, NIPCDDSFDQTDSQLWAILNKPSEPFELR, NLALFAEINELKK, NLISGQVELDTDTFYLPNK, NNAAYGNYEYLLER, NSDGYASLQNLIK, NSDYNLDLTVNVDHK, NVENIVFK, QIQLLNR, QLVEGYTLR, QSLLFEHSFVPEQTQNK, QTSDPHKQEISAAIQK, RVEFTGELTTPFDGYTNSK, SFLQTTPYK, SGIADSSSGSDIDIK, SGIVSTALNSCLQSSSAPVEIR, SILATGDIK, SQFFIVHNEVEK, SQFFIVHNEVEKEQEMIK, SSDIFINWDKDVATSNFR, STAHFLLNTLGSK, SVGMEQTFTNTDATLLNSGR, TGQFDFTLK, TPFEFLYDEINSK, PHVNYETLGLTMTHNPTAGALQSK, TTHVDVSSSPYGK, TYHAEFSWDPEK, VAHNHDEDDLTSSLK, VDNAEYVSTAVLDYSVR, VEFTGELTTPFDGYTNSK, VNDMVTGSASLALPGLQK, VNVMSFR, VNYFIPNLGK, VQAYSGTPYNFK, VSSAVTFNR, VSSSMYIYHPISLSNVK, YLTSTSQIK, YTGQLNWFIEDGEYDGK
BgApo2	1036	0.0131	2	FAFQDGR, TDLLFVHEEAEISSEK
Bg-Yolk- Ferritin	103	0.1692	13	ADVNLLGFK, ADVNLLGFKK, AGLYALEDTLALEK, ALDNALSVTTFINDR, AYASYFGR, DHVENFIDK, FPVVQLK, LEQQILSFHNQELALR, RNPADDNDNSDDDTRDNWR, SINNGLINLAK, SLTSYDILHPSFAASGR, TQPHICNFLTTSAVSK, VRFPVVQLK
Bg-Ferritin 1	161	0.1157	13	ADVALPGFSK, AYCLSNSAVK, DVNGEALMLHK, FFLEDGILSQK, GGYISLFDIPSPSVHETLLSR, LMSDLWQK, QNFYHMDELNTLIK, SFSDEDYPLGEYEVDLELR, SGLAGMESALDILK, SGLAGMESALDILKDVNGEALMLHK, SMLSYINK, SMLSYINKR, VQDIETIAQL***I***TR
Bg-Ferritin 2	877	0.0167	7	DVNTSLLELHK, ELADYVAQLTR, IAETNYDPHLDDFVEEELLGEQVK, QNYNEECEAAVNR, VGPGLGEYMFDK, VGPGLGEYMFDKE***T***LQK, VVLQDIK
Bg-Ferritin 3	423, 565	0.0436; 0.0313	11	DEWGAGVDAMQVALQLEK, EISDYITNLK, EISDYITNLKR, LASSHEDAQMADYLEDFLEEQVR, VGTGLGEYMFDK, VGTGLGEYMFDKESLS, DDVALPGFSK, DEWGAGVDAMQVALQLEK, IVLQDIK, IVLQDIKKPER, QNYHQDSEAGINR
Bg-Ferritin 4	877	0.0167	2	FAFQDGR, TDLLFVHEEAEISSEK

In some peptides, a single amino acid that does not match deduced amino acid sequence (Supplementary Fig. 1) is presented in a bold italic font. Peptides VVLQDIK and DVNTSLLELHK both appear in Bg-ferritin 2 and Bg-ferritin 4. The two proteins, Bg-ferritin 2 and 4 may be due to the high similarty of the sequences and low level of the expression. All peptides from proteins at the position 423 and 565 match Bg-ferritin 3.

Among the LLTP members, BgVtg1 showed the highest relative abundance. Although it ranked at the 11th position, the 10 preceding proteins are involved in cell structure, such as actin, myosin domain-containing protein, collagen alpha chain-like protein, ATP synthase subunit beta, tropomyosin, and globin, which are unlikely primary yolk proteins. A total of 85 peptides related to BgVtg1 were identified (Table 1; Supplementary Fig. 1). All the peptides are in a 1,660-aa N-terminal region (Supplementary Fig. 1). No peptides were detected for BgVtg2. Both BgApo1 and 2 ranked in the 565th and 1036th positions, respectively, in terms of their relative abundance. Besides, a total of 80 peptides were identified in BgApo1, compared to only two in BgApo2. Among the five ferritin-like proteins, Bg yolk ferritin was found in the highest abundance, which came in 103th position, followed by Bg ferritin 1 (161th). The abundance of the remaining three soma ferritins is low, at 423th or beyond (Table 1). The relative abundance (percentage) of BgVtg1 and Bg yolk ferritin proteins based on a total of the 4,729 proteins identified is 1.054% and 0.1692%, respectively, so there is sixfold difference between the two putative yolk proteins.

### Phylogenetic analyses of LLTP members and ferritins

**Figure 3:**
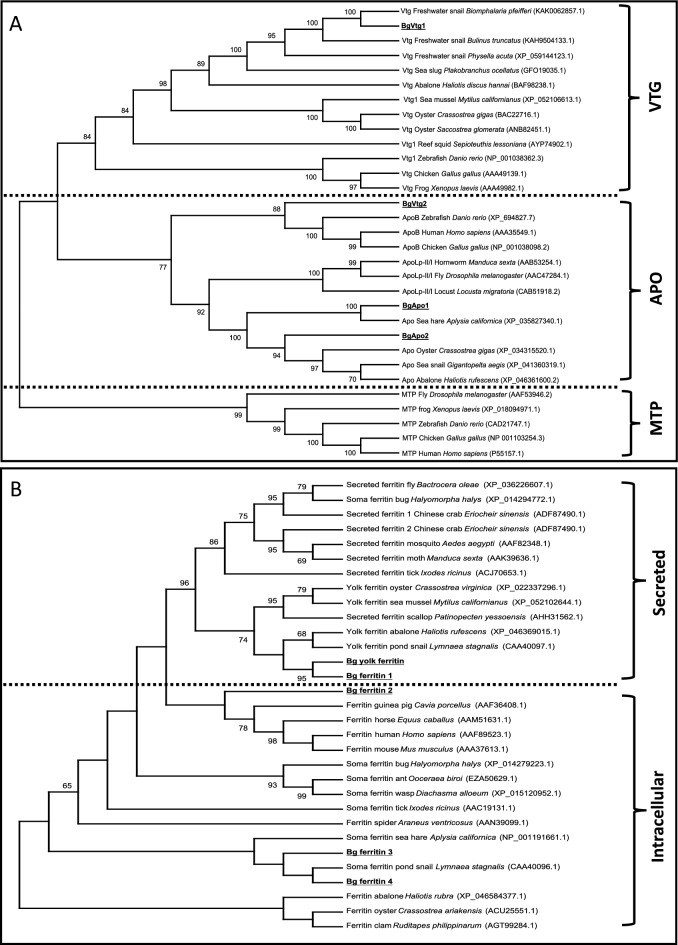
(**A**,**B**) show phylogenetic analysis of LLTP proteins and ferritin-like proteins, respectively. Bootstraps < 60% are not shown. The number in parenthesis is a GenBank accession number.

LLTP members were generally classified into Vtg, Apo, and MTP. Vertebrate ApoB was more closely related to Vtg than Apos from invertebrates such as insect ApoLp-II/I and mollusks’ Apos. As expected, BgVtg1 and BgApo1/2 were grouped with mollusk Vtgs and Apos, respectively. BgVtg2 was clustered with vertebrate ApoB proteins, again suggesting BgVtg2 is quite different from BgVtg1 (Fig. 3A).

For ferritins, secreted ferritins were separated from soma ferritins, but most branches had low bootstrap values (Fig. 3B). Both Bg yolk ferritin and Bg ferritin 1 were clustered with the closely related lymnaeid species, *L. stagnalis* (accession no. CAA40097.1) and the abalone *Haliotis rufescens* (accession no. XP_0463691015.1). This group was clustered with other mollusk secreted ferritins, forming a monophyletic clade that further clustered with insects secreted ferritins with a high bootstrap value (96%). Three Bg ferritins (2, 3, and 4) were grouped with intracellular ferritins and Bg ferritin 3 and 4 were clustered more closely with intracellular ferritins from the gastropods *L. stagnalis* and *Aplysia californica* than other species (Fig. 3B).

## Discussion

*Biomphalaria glabrata* is a hermaphroditic snail with both male and female reproductive systems in the same animal. The ovotestis comprises sack-like acini within which male and female gametes develop. Each acinus contains various developmental stages of female and male gametes and 8–9 types of supporting or accessory cells, such as follicle cells and sertoli cells^6,30,53,54^. Isolation of a particular type of cells, like oocytes and follicle cells, from the ovotestis for molecular studies has remained a significant challenge. As such, ovotestis has been targeted for the study. Therefore, it is not surprising that the MYP of oocytes is not the most frequently abundant protein in the ovotestis since the latter comprises many other germinal and accessory cells.

The structure and composition of the ovotestis differ from egg masses. An egg mass contains multiple capsules and each capsule contains a single embryo surrounded by yellowish fluid called albumen fluid which is derived from the albumen gland. Proteomic analysis of *B. glabrata* albumen fluid has shown that most of the major proteins in the fluid are defense-like proteins, which were thought to protect embryonic development^55^. The most abundant proteins in ovotestis, however, are involved in cell structure or to provide necessary nutrition such as yolk proteins for later embryonic development (see the Results section). Therefore, there is no major protein shared by the two proteomics profiles.

A total of 4 LLTP members (BgVtg1, BgVtg2, BgApo1, and BgApo2) were revealed based on the domain architecture. BgVtg1 was confirmed as a bona fide Vtg from domain architecture, high abundance presence in ovotestis, and phylogenetic analysis (see discussion below). The function of BgVtg2 remains to be determined based on its absence in ovotestis, expression pattern, and phylogenetic position. For the two BgApo proteins, more than one hundred BgApo1 peptides were revealed and matched perfectly to the deduced aa sequence, whereas for BgApo2, only two peptides have been revealed. Nevertheless, it is suggested that BgVtg2, BgApo1, and BgAPo2 are unlikely primary yolk proteins.

Importantly, our data provide considerable evidence supporting that BgVtg1 is a MYP in *B. glabrata*. First, BgVtg1 is the yolk protein with the highest amount in ovotestis, presumably oocytes. Second, the domain architecture meets the criterion of Vtgs proposed (i.e., SP + LPD-N + DUF1943 + VWD). Third, different from other LLTP members, BgVtg1 is well clustered with Vtgs from various animal species, and many of them were functionally confirmed as primary yolk proteins in these species. Fourth, the BgVtg1 expression pattern is consistent with other Vtg yolk proteins reported.

Although we could not investigate individual cell types in the ovotestis, we speculate that the highest amount of *BgVtg1* transcripts and its proteins mainly comes from follicle cells and oocytes, respectively. As far as known, yolk proteins are exogenously synthesized, secreted into the hemolymph, coelomic fluid, or blood, and transported into oocytes via oocyte receptor-mediated endocytosis regardless of the type of yolk proteins involved^1,4,24,56^. In invertebrates, the main synthesis site of *Vtg* transcripts is in either follicle cell or organ analogous to vertebrate’s liver (e.g., digestive gland)^1,4,57–59^. Recent studies in mollusks have shown that follicle cells are the site for the synthesis of Vtg in the bigfin reef squid *Sepioteuthis lessoniana*^60^ and the Pacific abalone *Haliotis discus hannai*^41^. The synthesis of MYP precursors in follicle cells was also implied in other gastropods such as *Ilyanassa oboleta*^61^ and *Aplsia californica*^62^. In the gastropod snails’ ovotestis, follicle cells surround oocytes. The information from our current study and published reports leads to the conclusion that BgVtg1, the MYP of *B. glabrata*, is synthesized in follicle cells and transported into oocytes through oocyte receptor-mediated endocytosis.

We have also investigated yolk ferritin in *B. glabrata* because yolk ferritin has been confirmed as an MYP in other gastropod snails. We identified a homology of yolk ferritin in *B. glabrata*. We demonstrated that Bg yolk ferritin is the same as the one discovered in the gastropod snail *L. stagnalis* regarding regulatory sequence, protein structure, and expression pattern (Fig. 2A,B,C). Bg yolk ferritin differs from cytoplasmic ferritins in structure and phylogenetic position (Fig. 3B; Supplementary Fig. 1). Importantly, our proteomic analysis does not support the idea that Bg yolk ferritin is a MYP in *B. glabrata* because of the limited amount of proteins present in the ovotestis. Given that its expression is higher than the four soma ferritins, we cannot exclude its role in oocyte development. Early studies have demonstrated that the secreted ferritin of ticks plays a vital role in embryonic development, but cytoplasmic ferritin does not^49,63^. We thus tentatively propose that yolk ferritin may serve as a secondary MYP in *B. glabrata*. Secondary MYPs have been reported in echinoderm animals and insects (see discussion below).

A profound difference in MYP employment between *L. stagnalis/P. corneus* and *B. glabrata* is very intriguing. As the discovery of yolk ferritin in *L. stagnalis* and *P. corneus* was made decades ago, Vtgs might have been ignored since then. To know whether *L. stagnalis* and *P. corneus* possess *Vtg* gene(s), we used BgVtg 1 and 2 as queries to the BlastN public databases of *L. stagnalis* and *P. corneus*. No significant similarity was found. Despite the finding, we still cannot conclude the absence of Vtg(s) in the *P. corneus* genome because sequence data for *P. corneus* is very scarce. For *L. stagnalis*, draft genome and a large amount of transcriptomic data have been published^64–69^. Moreover, Vtgs are large proteins (normally > 2,000 aa) and possess multiple conserved domains, making discovering their nucleotide fragments relatively easy. The absence of the Vtg sequences in the public databases implies that *L. stagnalis* may lose the evolutionarily conserved *Vtg* genes in the genome. Instead, it uses a non-Vtg protein (i.e., yolk ferritin) as a MYP^15,19,22–24^. This is unusual because the gastropod snails we discuss here are closely related, although the employment of different MYPs has been documented in some relatively related animals.

So far, four types of MYP have been reported: Vtg, lipases, ferritin, and transferrin^4,5,24,56,70^. In arthropods, despite *Vtg* genes in the genomes, higher Diptera (cyclorrhaphan flies, *Drosophila*) use lipoprotein lipases (unrelated to Vtg) as MYP. In contrast, in Lepidoptera (moths), Vtgs are employed as MYP^70^. A similar phenomenon has been recorded in the phylum Echinodermata. Extant Echinodermata comprises five classes: Asteroidea (starfish), Crinoidea (sea lilies and feather stars), Echinoidea (sea urchins), Holothuroidea (sea cucumbers), and Ophiuroidea (brittle stars). Transferrin-like protein, an iron-binding protein, serves as the MYP of the Echinozoa (Echinoidea + Holothuroidea)^56,71^, whereas in the Asterozoa (Asteroidea + Ophiuroidea), a sister clade to the Echinozoa, the MYP is Vtgs^72–74^. Again, transferrin and Vtg genes are present in their genomes but differentially expressed in different species^56,71–77^. All these studies lead to some intriguing questions. What evolutionary force drives those animals to selectively utilize different primary yolk precursors for vitellogenesis (i.e., Vtg, lipase, ferritin, transferrin)? How can ferritin and transferrin change their primary function from iron metabolism to oocyte development? Answering these questions will improve our knowledge of the evolution of vitellogenesis across the animal kingdom.

Concerning the gastropod snails, such questions become even more puzzling because of their very close phylogenetic relationship. Comparative investigations on yolk proteins and vitellogenesis of the mollusk species employing an ultrastructural, cell-targeted multi-omics approach in combination with endocrinological assays should advance our understanding of the reproductive biology of the gastropod snails, the largest clade of mollusks in terms of species^78,79^, which may help develop control strategies for snail-borne diseases^28,72,80^.

## Conclusions

Our comprehensive analysis combining bioinformatics, tissue-specific transcriptomics, ovotestis-targeted proteomics, and phylogenetics suggests that vitellogenin, but not yolk ferritin, is the major yolk protein in schistosomiasis vector snail *B. glabrata.* This study also highlights the need for comparative studies of invertebrate yolk proteins, particularly in gastropod snails.

### Supplementary Information

Supplementary Figure 1.

## Data Availability

The mass spectrometry proteomics data have been deposited to the ProteomeXchange Consortium via the PRIDE ^81^ partner repository with the dataset identifier PXD048379.

## Abbreviations

MYP: Major yolk protein
Vtg: Vitellogenin
RPKM: Reads per kilobase of transcript per million reads mapped
RNAseq: RNA sequencing
LLTP: Large lipid transfer protein
LLT: Large lipid transfer
LPD_N: Lipoprotein N-terminal domain
DUF1943: 1943 Domain
DUF1081: 1081 Domain
VWD: Von Willebrand factor D domain
MTP: Microsomal triglyceride transfer protein
ApoB: Apolipoprotein B
ApoLp-II/I: Apolipophorin II/I precursor
5’UTR: 5’ Untranslated region
IRE: Iron responsive element
TPM: Transcripts per million
