# Supplementary material for: Yolk proteins of the schistosomiasis vector snail Biomphalaria glabrata revealed by multi-omics analysis
Source: Sci Rep. 2024 Jan 20;14:1820. doi: 10.1038/s41598-024-52392-x (PMC10799875; doi:10.1038/s41598-024-52392-x)
Supplement: Supplementary file 1 — Supplementary Figure 1. [file 41598_2024_52392_MOESM1_ESM.pdf]

Supplementary Figure 1. Deduced amino acid (aa) sequences of 4 LLTP members and 5 ferritin-like proteins. Amino acid (aa) in red color are the peptides identified in proteomic analysis.

BgVtg1

MGDNYEILRKMLLPWIVALAGLTVTTIVAEREEYVYEYNTQILTGLPLHSSIHSGYRVNAIARVQFSTKSKVNVQLENIRLYNIRQPITQ 90

IEAHENLIPEEYLKELTGSESTVIVESLTTPFGFDYENGQVKRALLAKKEMEWSANAKRGFVSLFEIKLKTKRLLEDRQLLAGEDKSYTS 180

SYKVLETSAAGICSTLYTTECLSGNCSRQHVTKVRDYTQCLERPVYLQTKYDGFFPIGHSKDNPLTTGAVVKYLIEVYRGKPTILSAVAE 270

NRIVFSPYLNKGSVTTTVNQTLSLIQVKKSAIDIPDVFSLKVSLIMSLPSKSVDCQDPFELSKKSCKEPDLEVYSVDAIISQLKQAADNN 360

LNIASRESLVHLGVVVEMLSVSPRTVIKSLWEKLTHSLNQTEQHARKILFNVLPHVGTEAAVNQVLDICSQDLDFEKCAIAFNILTLKAT 450

PTETLINKFISLVKQQDKMSPVMKQTAYLSLGSLGYKLAQAKNRQLKELMKIEQLMLTLQTENSEALVQKKREVEEKRYKIVNSFDKLGK 540

NIVDTINELVVSGSKHSKILGLKALSNSGLPHAVPIFNKILANHEPHLYFRILAVLGHRRMYFDRTVREESLKTLLAVYNDKTEVNEVRS 630

WAFITLMQLHPELPVIQTIARNLNSEKDAYVSGLVLSYLSSHANTTFYPFTTFVKNCSDALKFAPRSNLNYQRSFTNLVTKMYDSAKIGK 720

VLSVTSIGSMNRFESWSFNLDTYVFGLYSNFLEVGINSMEVSQFLNKLFGPQGLLTMNKSIFDMLKRNKKSTSPQEMIEDIFKVLKIQSR 810

KGGMPAAHLYLRVMGHELRYLDLSEYISSLMANGKMSIYNNSSSVFPSLPLDVKTMYYLMNTKMTLPTEAGYPVSLKLKVSKTLNVKGSV 900

SLSVEPSIFEDERMGWPPKKVSASLDFRPKGVMEIYGSMGLDAHYMKRVASFRALVEFAMPIQKNISYDLSARKFKLVSIVPELNKPLVK 990

FLIQQYAESVTLTNNFTATHVDQLPLCAHKVIPINKFYDLNVLGYKVGFVGQLPYQFDWQNPLHIMGGKTYIRLMKKPMKDSPKVVTLTG 1080

QLVFPLEINSAAQNFSRDEVNQRVPISKVTNSFNQEETSYSDPVDLDKEAISMHQLINKFEDVTERTTIPLHHVPETRGIILSLDASTHK 1170

TVRKSQVEALWSRLDNGRIASAFIRLWKSPMPDYTPKEWQMVTEFRKTYPVKVHEPKDTLSATWQKEILRDLQYSVSKSSHQASNYLLHS 1260

TLLSEINELNYPAMMRTPTSRLLDNLVPEQLYMVMDKVEVSKNRWQKYWAKLVSLHEKVNEEKKRLVDPDDSKRDSNILCYINKVLKIQS 1350

TMNFDLDEIIKEKSVHCSELPIIQWIHFYYTTMINDLTELIPGYITKNIVLTQVQNIQNLMKQAKENQESIIKKMKQVFTLTLSDNEEKQ 1440

IKEKGNNFEMYKDYDVDQNAETDALYQVLAQLSLFQLEVIETLQKYNSRQQSCDIELIKCLTSETRNNITFFDQIWKDTPIYANNVIDTM 1530

LLVTIQNELIIHLLNVNSKLSLNTPITKSKEDTAKANSESFNLKIESLDLGQRVSSLNKQRMLLDVTSASLSEQELPIYNELKSIYRKLM 1620

VDRRIFEVNFAYNKWTHHDADVSETEVNKLLVSLNTYTTKLAELQKNVIDLDSTPNVYLATEFLGIIGSLRTTVDFCRPTLMAVTSPKKN 1710

IWIENFEAVSNNIQQLASVMSLIFDKFVTRTDEDQQTPLLFHPLDQTYQDIQKVTYDQDLLYERLTRLVQDNDSANFKLPQLISEIERLQ 1800

ELLKKTEPFLLDHLQHKTTTLSFVSRYLQAFKKQRKIYQIIQTTLEDKNGEDENKVLAKFNELEKLHSKMEVQINQLLSEKKSSNTYQSE 1890

NNAHKMSDPDEDTHPDLSSDNLPTYFSVITGTLNVSWGYTKRLENRFEVQMIATRSVQQLVHLYKQIDSKVRSTNVENAINDQLAGETWR 1980

YEKVMRRMNTYNHLHLTALYDKKVLPPWMLQLSQCLHESLKYYYWNHYTRHEPIESVSSDRTDVIVDLSNSYKDMNMFLMTGEETNKFLN 2070

IPAPYLLNKLKFNQNYKTIYDELTSSLSGGQTPAQCIISQESIRTFDQVTYPIPDTLGSCRAILAMDCSDEKTFAINMIRANSSSMSNAF 2160

QLLYESIEVEIQPSIVDILVKVNGNIIELEENKPVSFTKLLQYQRTEKYLIITKKDLGVHFSLPLAGISVHMDSLLLKIQVSELFKGRLC 2250

GLCGNFDYQVSWEYEGPSYEIYRTPKSFTYAYVLPDQTCPIPKDYKSDVEYYPPEKIVDLSQGIKRLKINKQLSNKSKVKKFKSRKSKII 2340

QKLFKN 2346

BgVtg2

MATSNGLNGYIFVYIYLVASLFRTSHVWAHECKHEGSCISPPKVCSNEGSRFRKGDRYEFSYEVMTSTGLTRDPHGTAGTKLTCNLVISV 90

LRKCELTMELLNCHLDDRGPSRFYTTTENSETLAEELSRHQVYFHYNEGMVIGHEIYVSELEPLYILNIKRGIVSLLQLPTKPDDLENKS 180

VELHDIFGTCLTDVTFPENTATYVQTYRDLVACNLSHISESQANVLSYSKTLIGGYRMAPITEFSYPFESEVTCDFELGKHNILKLARCV 270

QRQAHIPLGYEDQHFHSVVTNISQVIKFLSKTRLGNRPVDHSLNGKRLVKLTMEYESSDKKHSSDDLERELDVVLQDFIAQGHSNKINLD 360

HSAQSYRRLVHTLRDMEEDEIRKVKDAIFACRHSTLCYKAFNEKLDYKTLQSTKEGLLISSLMSCGSRDCMIFVAESLKLKHIERLEADR 450

ILLNLGLFYHPSSRFVKHIFDYCKHDYSSECLLAMSAMMSRLNDSLESTSHKQVVGYIVFDVLTYLHKLIKENGKPLIENRLNHRDKLIK 540

EETLLTAIKCVGNIGMYADKYYDSVLEKDLKITKYLLSLVKNQNISVSVARASVQALAAVGLKDAMINELLTVLKEERPPIVLRAAIFDE 630

ILIKGNETVMMKLVKLLEETSIENLQTYIVTKVSSLNEGLHLNHEEWRHKLTKLVEGLQIDQEENIWSKSQFSQFSHTVVIPLTSLRLGG 720

QLALNILYTPTSSLFHSFTIKGTLEIGEEVWSVFDIVVDIEGMDDIVHLLRSNNKNKTDKSNFIQEIMSLIQQKFREAGKSSTFHFKHFS 810

KTITQAIISIITSSNLQVTTLPKAYIFLKLNGYDVGWVSLNSFLTTLSHMKTSDSLVTTLIKTVTKICNTFLPQAVQLFDFHMTLPTIAG 900

YPLNTNLEAAFVAKSGLQIKHSPAINKLSLNLAFHPSITTHFQGGMQVSFGGYSRSKITSKISGFATLDLSTNIEYSTADTVAERELFLN 990

LVWNIGSEQFKFFNFKTDLFLLHNDIEHKVSPPETEKKFFKCLPDIYNLITGRRFCFHGSYFDVSKSGFYNFMNSFRITGTSDSEDEGLN 1080

QYTVNGSWSSNLQGPPEHQFRIQMSGLGSELSRILNITVNVDHHFGFKLQCFIPDANIVYVLQRKKVVNSTHHHIVYASVLQQESKTLYT 1170

LLFESKGHRIPKIGILPGNIILPYNHTDNVIHFSFALPSLSLDLRYKDRIHSATYATKDFIVKYFCEPKLKFLYAFHPVIRQAVSHGHTS 1260

TFSYHQEIMDSKTPLDKIWKAFYKFVIIWPEQKIVTTTEMVANMFSADRVSHLTWKYGNEVDEIHMTSHVTNKTTSHYQLFDYTLSLNNS 1350

GKFAVGLVGHFLIKGAKPFVNVTIRTDVKYVKTGMCEMKIKKTKSSCHSSDSKEKPLSIYDVIQVNMIRWLRGCDRAFELNNCQKWLEKE 1440

RFLQWWKGCELPVQEDNCKVTQGTEESFSLKNSFWTLDTWSYGMDGLILTKPHRTKSVVGQEAGLMLLWETNSTWPKAEEPFIATNGSLF 1530

MSRKQWVKLNLNDYVHNMMFTFQGEHLNTPQVFQHDHSWTLVETSEHSFDRSFKIHIDFEHYKHFDYNLQLCSPRSNVTHYINYTKISDL 1620

EHEIRANASIHSHQPKFKLSYFEATDINAVNFATHIDLMSTLLNYDLNLRKQTDANGLTMSANISADGPLLGAGSRKTFSTSIWLQKNHS 1710

PHWDLTYWSDKHSGIAADISLIKQNQMKLILNLLRIDQDKIKYIPFITWTLELVEPQKLHHKLEFSSLTAASIQHKMAVVQNTVSHVMRS 1800

SGQTMAHNIKAELKNKLLPILNESLDGFFKRQKIGTMAVDVLNELWLDLPLVQALKSSHIDELLATSVQYSTQFLSHWIVHPPFFINQFI 1890

KVHMYQNFLEALADKTLTFSQHLFVNMPTFMQWPKLPVFEKKLLAYIKYKTLGSQLKDKDVAVIIDGRRIKTFDGIIYDITEPPAQFCSY 1980

LICSDLKKGHFALTYAKAGLTLSLQKFGLTLAQDGSIISFGSKKIDYLPYYSNDAHLILSIHDGEFHIDIGSGLVHLIFNPVKSFYILAI 2070

DRTLNNATIGLLGTNNHEKGDDFLLINGTVIENSIDFQNNYELSKSDECHISEPPSSISCMPSNDQTCSVFSYPDLQVCNTILSSQPFKL 2160

QCQVDVCQKQSECRSIAAYVAACRSRGIYIHLPPHCECGNKLEGQEIVVVQSLHQKLIKSNDPLLSVKHLLSSFEHDASIGLVTYGESGL 2250

WAEPKAHLLDGKLLVKNTKALHNLPSAVSPGQNETSAEDAVKVAASYPFQSLNSKMIILIYHHVTGQKRIKETLLKTLQRKGIILVAVST 2340

YSTLEDKQIVGLLTDGKTIGTSASVTLPQDLFTTAVQESKGMWISLDTLIAGQPNNIRKIVYELTKLRSDSHNCTLSNDSHS 2422

BgApo1

METKLLICLALIASSNAGPTWRSNNDDDSSCALECKNNAR**KFQYAPSTTYIYDYVVDTETTMAGATEDSAK**LSIKTQAEIQSIDGCEFAL 90
SLKKTR**ISHSEAFAK**DLKAAEREDEFR**QLVEGYTLR**FSFDDGIIASVCPDEYENTWALNFKR**GLLSAFQNSMANLDSNDR**VTEIDVTGEC 180
KAEYNVSKSWGAWNKDTTVTKSKNLRACTNRDGYK**SFLQTTPYKIISDIQSLPIIR**STHMCSQVISSAKLLKSVTCEEK**HIYVPFSNDQS** 270
GGKTIVRQSLILAAQKSTVDAGRTYVSSR**QSLLFEHSFVPEQTQNKMESATQTLK**EICEQTVEDLRPETPR**MFSELVTVLR**MLDSSNLKH 360
LHRLLKDKKLCPENGRTIKFFLDALPMAETEGAVDLMTTLIINKDVEGLLAKAWMTSLALIQNPSISMIESALGLLDSNYDDAAFPVSSL 450
VNNYCKKHTNCGNTNGVIEVVKKLTSKIQRSCYVNEKNAGDVMKTLRALGNTGR**SGIVSTALNSCLQSSSAPVEIRAMAADAFRNIPCDD** 540
SFDQTDSQLWAILNKPSEPFELRIFSYLAVMRCPSSDNLDK**IVQLLEEEKDEQLGSYITSHLNNLKQTSDPHKQEISAAIQK**LTYKEFTT 630
SALKFSKNFEASFLVNK**LNLGLVADSDVISGLDSPLPR**YAKANFSLELFGNSINILEMGGRVEGVEALAEK**LLGPYFSGNK**DKSKDKVKD 720
IKGFAYAR**LFGNELFFQHFSGVEGLLK**SRNVPNFLDYMIQLAKRQEVAISHSQQLMDVSLVIPTIAGLPLNISVNGSINFDLKAEGKADL 810
RQVAAAPRSLDIDGEFKPSAALEITGTMAVDGLVAKLGLRMRNTMHSSTGIKGRIHINKGR**ELSIEIEPPKDK**MEIFNAK**SQFFIVHNEV** 900
EKEQEMIKTNYKQYKWCTEILAVEFCGDVQFPNASTMADAPYFPLTGPVK**VNVMSFR**KDTHTAYKFYAQRTESKDK**STAHFLLNTLGSK**V 990
NR**MISADVTIGYAPK**QLDVIINSPWRKLNLNGGMETNKDIYSLVGSILIDDVDKYGFTTEMKMSKKGSSAVYKPLVQTTYKGNTR**NLISG** 1080
QVELDTDTFYLPNKVLMDLK**VQAYSGTPYNFK**TYIVNNNKEKRFSTELSYGSNNAYIVNIGTTYNGKDFK**NFQVNPEVKIQTPSAVLLSL** 1170
TGTGDYKAEKSLSGNLTLNILKQKPAQFSLSMSNGMWGKIDMKLK**SILATGDIK**IENNLKKSMQKTSSIK**VNYFIPNLGK**DSFKVDIKYQ 1260
DKSTKSFSKYTLSSKTSHIK**NSDYNLDLTVNVDHK**DKRSQAESIIKYGADPKNTKKIINLSYVFKMDSFSLKDADIDLQVSATVPEKNIE 1350
SNLK**VAHNHDEDDLTSSLK**LKLLPYIKNNIAVSVNLKNSNQKSTKLQGSADLEFSGKKFTFNTDLNQKLKNQYVHTADFSGTSLGTHSIT 1440
TVYNIK**NDAYDVISDVNLDGYDPIK**LRGNLSLGSSNYGASLNGAYGR**DEYGVTVQTK**YIENRFGKLDLTLVHPDRQIIAAGEAKILSGKY 1530
EGSALVNWNAAKKNTKDQASVEGIFSNIVKGESTVISGDVKIK**TPFEFLYDEINSK**VVLTSDAKKISTTSRLSWGKLQR**VSSSMYIYHPI** 1620
SLSNVKMNLNVETPYRAMK**ELNFEIDHTLDTDLSTTVR**GKLNDDKGELILIGENKGSSYKNDLSTSLNLKTTVPLFNDISLALSHSDDSA 1710
NYQSSGTFSYNGDKYETAITMSHSLIGYNVKNSGKLR**MSWPTDSLETTWTHDNTDTR**RMNCAISTTWNNNRKTMTVTFDGSNFLSRGERQ 1800
SKASLEIKTPWQEVRNLKVDMNQQHSSMRLLHKLDIKK**NNAAYGNYEYLLER**DTGSINTKFLVKLPQVDTIHGQTTVKYERFPASTSFWL 1890
EWAPSSR**IDFDGSLNAPSIELIELDTK**LSTPFR**NVENIVFK**VSHK**VDNAEYVSTAVLDYSVR**K**QIQLLNR**FQLDDNIIAWRTTFTTPCKH 1980
FKLLNYGLKVDMTDDKFDGSADFELQPLVQK**YTGQLNWFIEDGEYDGKLTLNTPLR**NLPDFQITSWRKEQDDGSK**TTHVDVSSSPYGK**YM 2070
YDATYTTEAPYSLDIQIK**TPHVNYETLGLTMTHNPTAGALQSKLDLIYLMK**KELTVDLNLNWKNKIDVSLIILNTPLKYYRNNR**LTFSHET** 2160
TRSGFLCRIDSVFGVSPLKGDASYAR**VNDMVTGSASLALPGLQK**FEGKFKLTGGLKDMKGDANVK**LGYNTVSTDFSNLMK**SNLYKTTFNL 2250
YTPYTEDLKVEFKHDDKSNR**NENKFDTSFNAQYGDYER**SARLSVSCSGLK**TGQFDFTLKGPSDSLVVSLNHEITGDK**VVGSSKIESSIDS 2340
IGNIEFSLAKEGSLQDMTASAKLVRNREQLVDTSVQNKWDNTVTGKFNVRGKWVPTIQVLHDHKGSYSNFNNKVRVTVDGKVVLDKELSA 2430
KFDDKIMDVTGK**MNYALGTEEHK**HSLSIHKEGDFNNLKLTINKKCDGQESKITIELQTDDQVKNLVHIENFMDYGFIGYELEHSGDFDKF 2520
LTNVKVYMTKDDIITGRVDFFKYSTR**RVEFTGELTTPFDGYTNSK**IEYRHEGQDNRMSCNINLKYGDSKIYSSDLIIQREPSMSLSFNVK 2610
TPYRGYEQLLLSSNFDGKTFNGHAILGRGREIKTSGVSGSQYELTTTFDQLKQVTINLDGKLTDGSADFVLLAGNKRMSGKAKVLYNNMD 2700
SLSASAELVSNVKNLEALKFLVKKAASAGSK**TYHAEFSWDPEK**QVVIDSKYSNSNFARSLKK**EHAFELDIK**LPYEALREINLDVNYNSVG 2790
QNALGSK**LSLDVNQDKIIDLDVDYR**NSDQHEVSVVVR**KPWPQEYIVIADLKEDKSSDIFINWDKDVATSNFR**IK**SGIADSSSGSDIDIK**M 2880
NMEVTYRSR**SVGMEQTFTNTDATLLNSGRIYWDNKEDSR**IMYSININGGSSSNTREFKVGFPSRTLILKGSSSRVGSTQNVDATFGWDMD 2970
RDDTKVIGLTASVYNQDYTQADITVKMPSDLK**VSSAVTFNRGNTIFEGTTAFSYNSDSR**KTLTLFSTVKNSPYATRRVFQSAENNYNYSV 3060
QLGVKHPETKVDIKIDSHLGKRANVMTSSMGVR**YLTSTSQIKNLALFAEINELKK**EMNLVAATSSNNVGLSTRIINDEPLQVELATTVNG 3150
QQSFKSMVDSTDRAVNLIIGEVSFNAKYNNASSVTFEINMLNDGNK**ISDAALTFTLVKPK**LLQSSLIWKPKLLANFRKWLINEAVRNADD 3240
FKVFLRQLNSEVRNELVAKHR**DISSFFVETLK**LAAKDAETELNSLAVSMDQLQQSLTLLRR**NSDGYASLQNLIK**RIQYDFQKLIQSYKSV 3330
YRNAISSITASLDQVKQYLVAINYRETVTDTMETTFRVMDNVIIAFTDFIQKDIDFSPMKSLSDKAMQSTEELKKELESIKKTVLNLDLG 3420
DIVTRKLSFLNEYKTSIENVQKK**IISQVPEQLK**ETLQIIFGELIHQVDYLSRYLEIKSTLKNHINFAIQLINDIALEDLTEFIDNLKEML 3510
KDPIVAFDLENGR**IILNVPLPMALER**LDHLPK**LSDSINNANQFIQK**YLPNRESFPNIYQYVWNGKNRTDDIIKTELNDFEPLRRRILPSR 3600
YRNVILD 3607

BgApo2

MEAYTILLLLIPVVIGGPIWLEEDLQNDPIISSYDCSASCTGSNKFNYEVGKTYIYDYEVATATNMQGASESVAKLDVKAKVTIQVLSKC 90
EMVLSLSEVAVQRSDPDSTTMVLAEDQTFKLALERNSLR**FAFQDGR**IDTLCPSVGDEVMSLNFKRGVLSVFQNSMNQLQNGENLQEIDIT 180
GTCPTAYTITQKGWKSTEVTRTKDLLGCSDRHNYRSALKSTPIHVPDTIKSLPLLKGSYKCSQMINSEGLLSTAICTESLVFRPFSKKES 270
GARTETVQKLTFVSKLSSVNTRKDLISSK**TDLLFVHEEAEISSEK**TLWSAKDKLTQLCRITAQDLPVEVPDLFTDLVYALRSLKSSLMTD 360
LYEQLKRGSICSDNKERIKKFFLDALPMVETSASIEVMGQMLVSEDVSGSQADYWLNSLHFVRKPTSDMISQLLTIVKTDKLQLKSLLPV 450
SSMIKTFCQSNEDCVSNRDIGDILNVWENILDDKCQSTDEKLVLMTLRAAGNMGYSDKLARKLKECITSQETAMNARVFAAQAYTGISCS 540
ADRQVLMKTFEDVKEDSELRIAAYLSAMHCWNEELLKKVKLVLESETSNQVSSFVWTHLTNLMETSSPYKQALKDILNDEVIKKKSGTNS 630
KHSGNYEVSFFSERFGAGATAESNLIWSTKSFLPRSAMVNLTVDIFGQSVQLLELGGRVQGIEALLQKFIGSKLFKGGESVTTDSGASGC 720
VENDKIENMKKKYPSEQDMLTASLYLRMFGHELDFKHYNEKELLALRDQVPGKVTSKGVNQDISYSQSFMFLESQMTVPTIAGLPLTLSI 810
NGTASLDLQAKLESDVKKHTMKATFDIKPSGAILVSGLMSINAGPTSTGLKMTSTLHTSTSVTGRIEAGDKQVLSLEFDTPDKVMDILEI 900
KTDFFILHNKNEKKQSMLTDKKMTMTSCTPDSVFKVTGYELCAAVAYVDSTKNPSTPYMPLSGPLLLSLTLQKKDAPKGYKMTIKYIQNV 990
DTSTASVAIDTSGSKVDRKINIGYYLDTKERKLDAEFVSPWKTIMFEATYPQERSNMHAFSASANVDGDLYKIFWQADKKLMKGKEIWSP 1080
KLEIMRPKQSRLSSYSPESVSARGTVEINEDLEEISTDLTANIVSNDQTALGKTLKALFRDGYVALKAKYINKEKQKTFSGSFSLNKKDS 1170
YSATLQLLLSDKKSGTNKYEPTILISTPENEKLFLLSGKMDYKPEKSFDGNLQINMDQTLKEPITLN 1237

Bg Yolk ferritin

MILYILVMFTLIGSSSQQDLGFVRHVSQNFHEKINSKLEQQILSFHNQELALRAYASYFGRADVNLLGFKKLLTGLRDRALDNALSVTTF 90
INDRGGRVRFPVVQLKDACNEITKSLTSYDILHPSFAASGRTQPHICNFLTTSAVSKKRNPADDNDNSDDDTRDNWRAGLYALEDTLALE 180
KSINNGLINLAKEATKFSDPQTRDHVENFIDKQVEVIQKLAEIITKLRRYQQDDEYILGEYLINNELES 249

Bg Ferritin 1

MNLTPGCEKQLQGCDDFKEPKQRLGSMQCKFYKSSCSYSCTLRCPRKDGKFLQISDKRYAKETFNCIAGVWKRKETPKAYCLSNSAVKEV 90
RQNFYHMDELNTLIKTLFNTSYNLIGMATFYERADVALPGFSKLMSDLWQKEVQLARSMLSYINKRGGYISLFDIPSPSVHETLLSRLES 180
KSGLAGMESALDILKDVNGEALMLHKQATDNKKMSDPHLKFFLEDGILSQKVQDIETIAQLTTRLKSFSDEDYPLGEYEVDLELR 265

Bg Ferritin 2

MSLARQNYNEECEAAVNRQINLELYASYVYLSMAYHFDRDDVALPGFHKFFKKMSQEETGHAELLMKYQNNRGGRVVLQDIKKPDTDDWG 90
NGLAAMTTALKLERDVNTSLLELHKIAETNYDPHLDDFVEEELLGEQVKSIKELADYVAQLTRTVTESVLKMCSRVMYIMDDDEPFVPVA 180
LHQDSNAESGISFQNHSDVNKTPLSSVVCEKSTDHSLFKDLGSLSVEHLPKSYQHEKILSLIKSVADLAVLIHVPVLNQAGLYWQGTGRV 270
EDAFIFKENTICQCKKCQLSENPSKAWGKVQIVTSTKLLTACQYVDEVKCTLFHDDEEKTENLAYLFGDAYIFNEGGVCKFYCYTCDMDL 360
LNKLDAYLDTFESKWISAFVKYYETVKSAEERLLVMICHPHGCKKYVSIGKWCKLRPSVTSCPGCEGSFLLIPNFDVNDDDYC 443

Bg Ferritin 3

MYAVGGINSRGRRQETSVVCLATSVNVQANLNVSKYSTTATAVVAALPSPASEPIVPSDRIPASPSPAFKTVRVKEASTCAVMSLHLSQC 90
RQNYHQDSEAGINRQINMELYASYCYQSMGFYFERDDVALPGFSKFFKKLSSEEREHAEKLMSYQVKRGGRIVLQDIKKPERDEWGAGVD 180
AMQVALQLEKSVNQSILDLHKLASSHEDAQMADYLEDFLEEQVRSIKEISDYITNLKRVGTGLGEYMFDKESLS 254

Bg Ferritin 4

MSVSQCRQNYHADCEAGINRQINMELYASYTYQSMAFYFERDDVALPGFHKFFKKQSEEEREHAEKLMKYQNKRGGRVVLQDIKKPDRDE 90
WGTGLEAMQVALQLEKSVNQSLLDLHKVCTDHEDPQMADFLESEYLEEQVESIKQIGDYITNLKRVGSGLGEYLFDKETLGSD 173
